# Autogenous bone grafting does not enhance bone repair in maxillary sinuses grafted with β-tricalcium phosphate: a split-mouth randomized controlled trial

**DOI:** 10.1007/s00784-026-07081-6

**Published:** 2026-08-22

**Authors:** Luiz Cesar Peruzzo, Albert Sabin David da Silva Ferreira Corrêa Souza, Luisa de Lanna Reis Rocha, Andrei Correa Guandalini, Rafael Silveira Faeda, Rogério Margonar, Guilherme José Pimentel Lopes de Oliveira, Elcio Marcantonio

**Affiliations:** 1 Department of Health Sciences, Implantology Post Graduation Course, Dental School, University Center of Araraquara (UNIARA), Araraquara, São Paulo, Brazil; 2 Department of Periodontology and Implantology, Dental School, Universidade Federal de Uberlândia -UFU, Pará Av. 1720, 38405-320 Uberlândia, Minas Gerais Brazil; 3 Post Graduation Course in Implantology, Instituto Latino-americano de Pesquisa Odontológica (ILAPEO), Curitiba, Brazil; 4 Department of Diagnosis and Surgery, School of Dentistry at Araraquara, Paulo State University–Univ. Est. Paulista/UNESP, Humaita 1680, 14801-903 Araraquara, São Paulo Brazil

**Keywords:** Bone augmentation, Calcium phosphate, Histological analysis, Sinus floor elevation

## Abstract

**Objective:**

This split-mouth randomized controlled trial evaluates whether the combination of autogenous bone (AB) graft with β-tricalcium phosphate (β-TCP) provides advantages in bone availability following maxillary sinus floor elevation.

**Materials and methods:**

Fifteen patients underwent bilateral maxillary sinus floor elevation surgery. The sinus cavities were randomly filled either with β-TCP or with a 1:1 mixture of β-TCP and autogenous bone graft. Ten months after grafting, dental implants were placed. Implant stability was assessed through insertion torque and resonance frequency analysis. Histomorphometry were performed on biopsies obtained at the time of implant placement. Cone-beam computed tomography was used to evaluate bone availability.

**Result:**

The implant insertion torque was 37.71 ± 14.52 Ncm in βTCP + AB and 41.88 ± 15.80 Ncm in βTCP grafted areas. Sinuses grafted with β-TCP + AB showed 43.75 ± 13.67% of bone while sinuses grafted with β-TCP presented 49.32 ± 13.41% of bone. The linear height in sinuses grafted with β-TCP + AB were 3.38 ± 1.56 mm at baseline, 14.18 ± 3.54 mm at 14 days post-surgery, and 12.12 ± 3.54 mm at 10 months post-surgery. The sinuses grafted with βTCP presented linear heights of 3.17 ± 1.51 mm at baseline, 15.62 ± 2.41 mm at 14 days post-surgery, and 11.81 ± 2.12 mm at 10 months post-surgery. The use of β-TCP alone was associated with greater graft volume reduction than β-TCP+AB, and this difference was more pronounced in sinuses with lower residual bone height.

**Conclusion:**

After 10 months of healing, the addition of AB to β-TCP did not increase the amount of newly formed bone in maxillary sinus grafts. However, β-TCP alone was associated with greater graft volume reduction, particularly in more atrophic sinuses.

**Clinical relevance:**

The addition of AB to β-TCP did not enhance histological bone formation after 10 months of healing, although it improved graft volume maintenance, particularly in more atrophic sinuses.

## Introduction

The use of dental implants for the treatment of edentulism is a routine procedure that has shown favorable outcomes in a variety of clinical situations [1–3]. However, certain factors increase the complexity of this treatment, such as limited availability of the bone required for proper implant placement [3, 4]. When considering these clinical scenarios, rehabilitations in the posterior maxilla deserve special attention, as this region is particularly prone to insufficient bone volume due to the reduction in alveolar ridge height following tooth extraction and the concomitant pneumatization of the maxillary sinus [5, 6]. Consequently, techniques for maxillary sinus floor elevation combined with the use of bone substitute biomaterials have been efficiently applied to overcome this limitation [7, 8].

Among bone substitute biomaterials, the autogenous bone graft is considered the gold standard [6, 9], as it is the only material that exhibits all three biological properties of bone formation—osteoconduction, osteoinduction, and osteogenesis [5]. However, due to several clinical limitations associated with its use, such as the limited amount of graft material available, donor site morbidity, and high resorption rates [5, 6], synthetic biomaterials have been proposed as alternative materials for maxillary sinus floor elevation procedures [7]. It has been reported that despite the favorable biological properties of autogenous bone grafts, osteoconductive bone substitutes have shown promising clinical outcomes and represent a viable alternative to the use of autogenous grafts [10].

Beta-tricalcium phosphate (β-TCP) is a synthetic biomaterial with osteoconductive properties that has been widely used as a bone substitute and shown favorable clinical outcomes in maxillary sinus floor elevation procedures [5, 6]. However, the absence of osteoinductive and osteogenic properties in bone substitute materials is associated with limitations in the promotion of the bone healing process [11]. Therefore, several strategies have been investigated to enhance the biological potential of osteoconductive grafts [12–18]. Clinical studies that combined bone substitutes with growth factors such as recombinant human bone morphogenetic protein-2 (rhBMP-2) [19] and recombinant human growth and differentiation factor-5 (rhGDF-5) [14] demonstrated increased bone formation; however, the therapeutic application of these growth factors remains costly [20]. An alternative approach to improving the biological properties of osteoconductive grafts, which has shown promising results, is the combination of these biomaterials with autogenous bone [13, 15, 16, 21]. Nevertheless, to date, the combination of autogenous bone and β-TCP in maxillary sinus floor elevation procedures has only been minimally explored.

Therefore, the aim of the current study was to compare bone healing in maxillary sinuses grafted with β-tricalcium phosphate (β-TCP), associated or not with autogenous bone graft, in maxillary sinus floor elevation procedures. The null hypothesis of this study was that the addition of autogenous bone graft would not influence bone repair in maxillary sinuses grafted with β-TCP.

## Materials and methods

### Ethical considerations

This study was approved by the Human Research Ethics Committee of the School of Dentistry of Araraquara (CAAE: 32435614.5.0000.5416). In addition, the study protocol was registered in the Brazilian Clinical Trials Registry (ReBEC – RBR-7qhkrn - https://ensaiosclinicos.gov.br/rg/RBR-7qhkrn) on November 11, 2016, under the WHO Universal Trial Number (UTN) U1111-1173-6057. All participants read and signed an informed consent form prior to inclusion in the study. The fundamental ethical principles for research involving human subjects, as stated in the Declaration of Helsinki, were fully respected. The results from this clinical trial were reported according to the CONSORT guidelines. The patients were enrolled at the specialization clinic in implantology at the School of Dentistry of Araraquara.

### Eligibility criteria

The inclusion criteria for this study were as follows: Age between 18 and 70 years; need for bilateral maxillary sinus floor elevation prior to the placement of osseointegrated dental implants; residual alveolar bone height adjacent to the maxillary sinus equal to or less than 4 mm; good systemic health; at least 4 months of post-extraction socket healing. Patients were excluded from the study if they met any of the following criteria: Current smokers or those who had stopped smoking within the previous 5 years; diabetic patients; use of medications or presence of systemic conditions that could alter bone metabolism; chronic upper airway infections; patients presenting asymptomatic sinus opacifications detected by the image exams; chronic use of anti-inflammatory drugs or antibiotics; presence of bruxism; alcohol abuse; drug dependence; pregnant women or those planning to become pregnant within the following year; a history of radiotherapy in the head or neck region.

### Groups and study design

The maxillary sinuses of the patients were randomly assigned, in a 1:1 ratio, to be grafted with the following biomaterials: β-tricalcium phosphate (β-TCP; BETApro^®^, 99% purity and 20 a 600 μm of particle size, Procell, São Carlos, Brazil) or a combination of β-TCP and autogenous bone. Patients underwent the surgical procedure for the placement of the bone substitutes, and a 10-month healing period was allowed before implant placement surgery, during which biopsies were collected from the grafted sites. The implant prostheses were placed for rehabilitation six months after bone substitute placement. Randomization was carried out using a randomization table generated on the Random.org website (GJO). The operator only became aware of the side to be grafted with the different treatment combinations after performing the maxillary sinus lift (LCP).

### Sample size

Regarding the sample size, the initial protocol estimated the inclusion of 20 participants. However, during the course of the study, we recalculated the sample size based on the variability observed in the early data and on the specific outcomes related to the maxillary sinus. This updated analysis indicated that 15 participants would provide adequate statistical power for the outcomes of interest. The sample size was calculated using a paired t-test based on tomographic data on bone formation from the study by Stiller et al., [9], which evaluated the volumetric stability of β-TCP grafts and β-TCP combined with hyaluronic acid for maxillary sinus floor elevation. The minimum difference between mean graft volume reductions was 13.90%, with a standard deviation of 5.00. Thus, considering a study power (β) of 0.9 and α of 0.05, a minimum sample size of 15 patients per group was determined. The sample size calculation was also based on the number of implants placed, with implant stability quotient (ISQ) defined as the primary outcome. Based on data from a previous study, a minimum clinically relevant difference of 10 ISQ units and a standard deviation of 8.45 units were considered [22]. Assuming a split-mouth design, a two-sided significance level of 5% (α = 0.05), and a statistical power of 80% (β = 0.20), the minimum required sample size was calculated to be 12 implants. Therefore, a minimum of 12 implants was required to detect a statistically significant difference between the treatment groups.

### Surgical procedures: maxillary sinus floor elevation

Patients underwent maxillary sinus floor elevation surgery. Following local anesthesia, a full-thickness mucoperiosteal flap was raised to expose the lateral wall of the maxillary sinus. Using a round bur mounted on a contra-angle handpiece, an osteotomy was performed to access the sinus through the lateral wall. The sinus membrane was visualized by transparency, carefully elevated, and the lateral wall window was displaced into the sinus cavity. The grafts were inserted into the sinus in volumes of 2–5 g, depending on sinus anatomy and volume. One sinus was filled with a mixture of β-TCP and autogenous bone harvested from the patient’s mandibular retromolar region, while the contralateral sinus received only β-TCP. The mixture of autogenous bone and β-TCP was based on a visual assessment of the harvested autogenous bone volume. No weight-based measurements were performed. The graft materials were mixed intraoperatively to obtain an approximately 1:1 volume ratio before placement. After graft placement, the surgical site was covered with a resorbable collagen membrane (Genderm^®^, Baumer, Brazil) and sutured using 5 − 0 nylon sutures (Ethicon, Johnson & Johnson, Brazil). The donor site was closed in layers using 4 − 0 Vicryl sutures (Ethicon, Johnson & Johnson, Brazil) for internal closure and 5 − 0 nylon for external sutures. Postoperative medications were prescribed to all patients. Amoxicillin (500 mg) was administered orally three times daily for 7 days, ibuprofen (600 mg) three times daily for 5 days, and dipyrone (500 mg) four times daily for 3 days. Additionally, patients were instructed to rinse with a 0.12% chlorhexidine digluconate mouthwash three times daily for 14 days. Sutures were removed after 14 days.

### Surgical procedures: implant placement

Ten months after the sinus floor elevation procedure, implants were placed in the grafted areas. Following local anesthesia, a full-thickness mucoperiosteal flap was raised to expose the alveolar ridge for implant placement (Drive^®^, AQUA, HE connection, 4.3 × 10 mm or 4.3 × 11.5 mm, Neodent, Curitiba, Brazil). Implants were installed in the indicated regions, and for prosthetic convenience, some implants were also placed in native, non-grafted areas. During implant placement, biopsies were obtained using a 2-mm diameter trephine bur from the implant sites. After implant insertion, the surgical site was sutured using 5 − 0 nylon sutures (Ethicon, Johnson & Johnson, Brazil). Postoperative medication and care were identical to those after sinus graft surgery. Sutures were removed after 14 days. Implant prostheses were placed for rehabilitated six months after surgery. Of the 15 patients evaluated, 13 received full-arch implant-supported prostheses, while 2 patients received partial implant-supported prostheses for posterior segment rehabilitation. Neither the operator who placed the dental implants nor the patients were aware of the side assigned to each treatment combination after the maxillary sinus lift procedure (ASDSFCS).

### Implant stability analysis

Implant stability was assessed at the time of placement using the Osstell^®^ device (Osstell AB, Göteborg, Sweden), which determines implant stability through resonance frequency analysis by calculating the Implant Stability Quotient (ISQ). Results are displayed on the device on a scale from 1 to 100, with higher ISQ values indicating greater implant stability. Insertion torque values (Ncm) were also recorded during implant placement. Primary stability data were collected for all implants placed in the study participants, including implants inserted in grafted sites (β-TCP and β-TCP + AB), which presented ≤ 4 mm of residual bone height according to the eligibility criteria, as well as implants placed exclusively in native bone. Stability measurements obtained from implants placed in grafted sites and those placed exclusively in native bone were analyzed separately.

### Histomorphometric analysis

Biopsies collected for histomorphometric analysis were fixed in 4% paraformaldehyde for 48 h. Samples were then decalcified in 7% EDTA for 60 days and subsequently processed for paraffin embedding. Histological sections of 5 μm thickness were obtained and stained with hematoxylin and eosin (H&E). Images of the sections were captured using a DIASTAR optical microscope (Leica Reichert & Jung Products, Germany) with a 4×/10× objective. The images were digitized with a DXC-107 A/107AP video camera (Sony Electronics Inc., Japan) attached to the microscope and transferred to a computer. The percentages of bone, soft tissues, and bone substitute were determined in two sections of each biopsy. Quantitative analysis was performed using image analysis software (ImageJ, Jandel Scientific, San Rafael, CA, USA). The analysis was performed by a trained and blinded evaluator (LLR).

### Tomographic analysis

Radiographic images were acquired using a cone-beam computed tomography (CBCT) scanner (i-CAT Classic; Imaging Sciences International, Hatfield, PA, USA), generating DICOM-based datasets with a resolution of 96 dpi, 14-bit grayscale, and voxel size of 0.25 mm. The scanner was operated at 120 kVp, 5 mA, with an exposure time of 20 s. CBCT scans were performed preoperatively, 14 days after the maxillary sinus floor elevation procedure, and one week before implant placement. Using virtual planning software (DentalSlice Bioparts Software^®^, Brasília, Brazil), measurements of the maxillary sinuses were taken at the three time points, at precisely the same locations. The distal limit of the nasopalatine canal was adopted as a fixed reference point in all three scans. From this distal limit, slices were numbered to the central region of the grafted area, with two additional mesial and two additional distal slices, resulting in a total of five slices per maxillary sinus. This approach allowed measurement of the same region across all three CBCT scans. Linear measurements from the alveolar crest to the sinus floor (mm) were performed. Additionally, the percentage of residual bone in the grafted region after 10 months was compared to the bone height immediately after biomaterial insertion to evaluate the dimensional stability of the grafts. The analysis was performed by a trained and blinded evaluator (ACG).

### Statistical analysis

Statistical analysis was performed using GraphPad Prism 9 (San Diego, CA, USA). Normality of the data from resonance frequency analysis, histomorphometric analysis, and tomographic analysis was confirmed using the Shapiro-Wilk test (*p* > 0.05). Implant insertion torque data did not follow a normal distribution. Parametric one-way ANOVA followed by Tukey’s post-hoc test was used for comparisons between experimental groups for resonance frequency analysis, tomographic and histometric analysis. Non-parametric Kruskal-Wallis tests followed by Dunn’s post-hoc test were used for comparisons of implant insertion torque (βTCP vs. βTCP + autogenous vs. Native Bone). Linear and volumetric bone formation and the composition of the grafted areas were analyzed using paired t-tests. Additionally, a Pearson correlation test was performed to assess the relationship between graft stability and the residual bone in the posterior maxilla. All tests were conducted with a 95% confidence level.

## Results

**Fig. 1:**
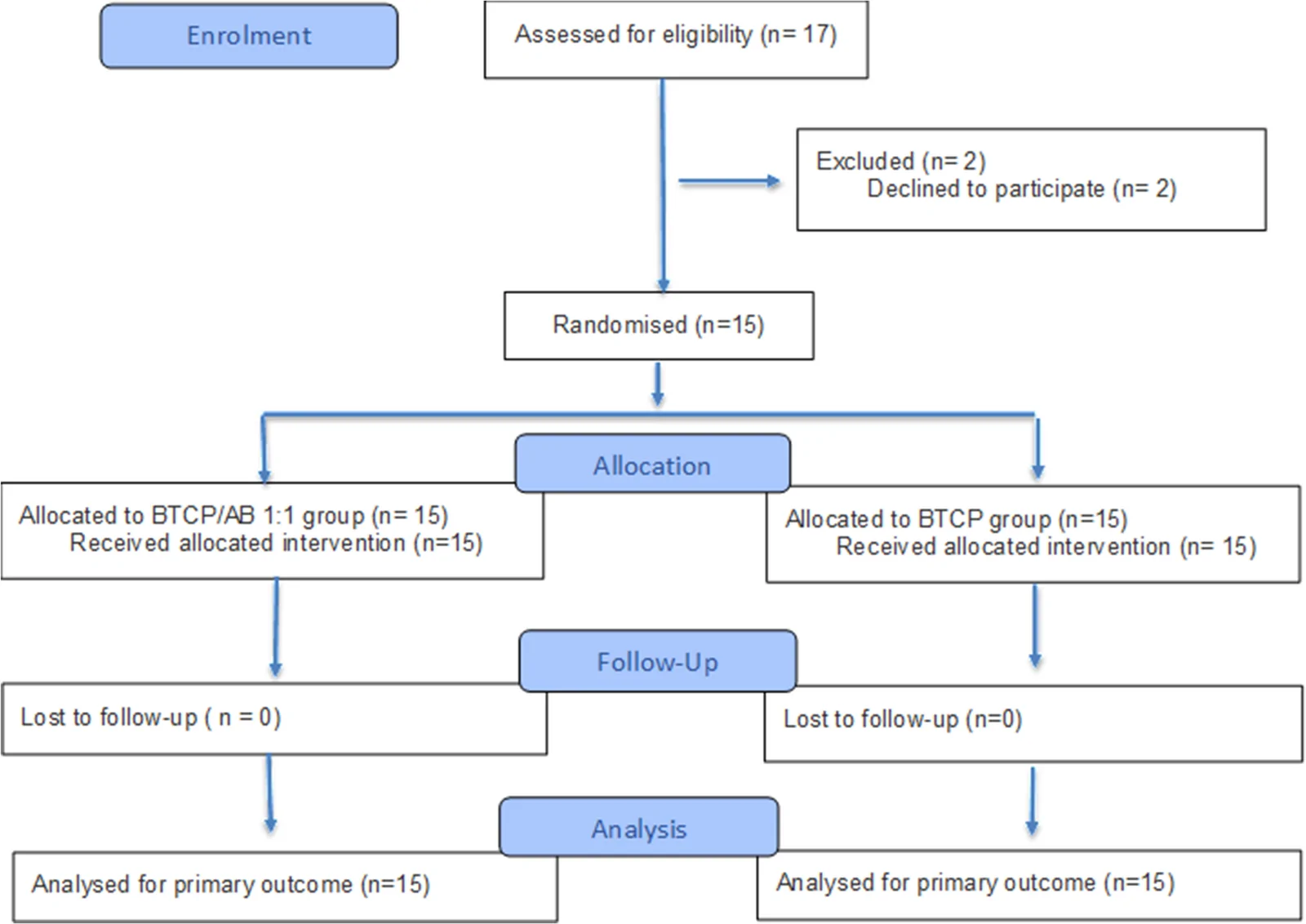
CONSORT 2025 Flow Diagram illustrating the process of participant selection, allocation, follow-up, and analysis in the study

Patients were surgically treated and rehabilitated between January 2015 and March 2017. A total of 75 implants were placed, of which 33 were installed in sites grafted with the combination of βTCP and autogenous bone, 31 were placed in sites grafted with βTCP alone, and 11 were installed in native bone sites (Fig. 1). Membrane perforation occurred in three maxillary sinuses; however, these events did not compromise the surgical procedure or result in any complications. The perforations were isolated and covered with a collagen membrane. All implants were successfully placed without further complications.

### Implant stability analysis

**Fig. 2:**
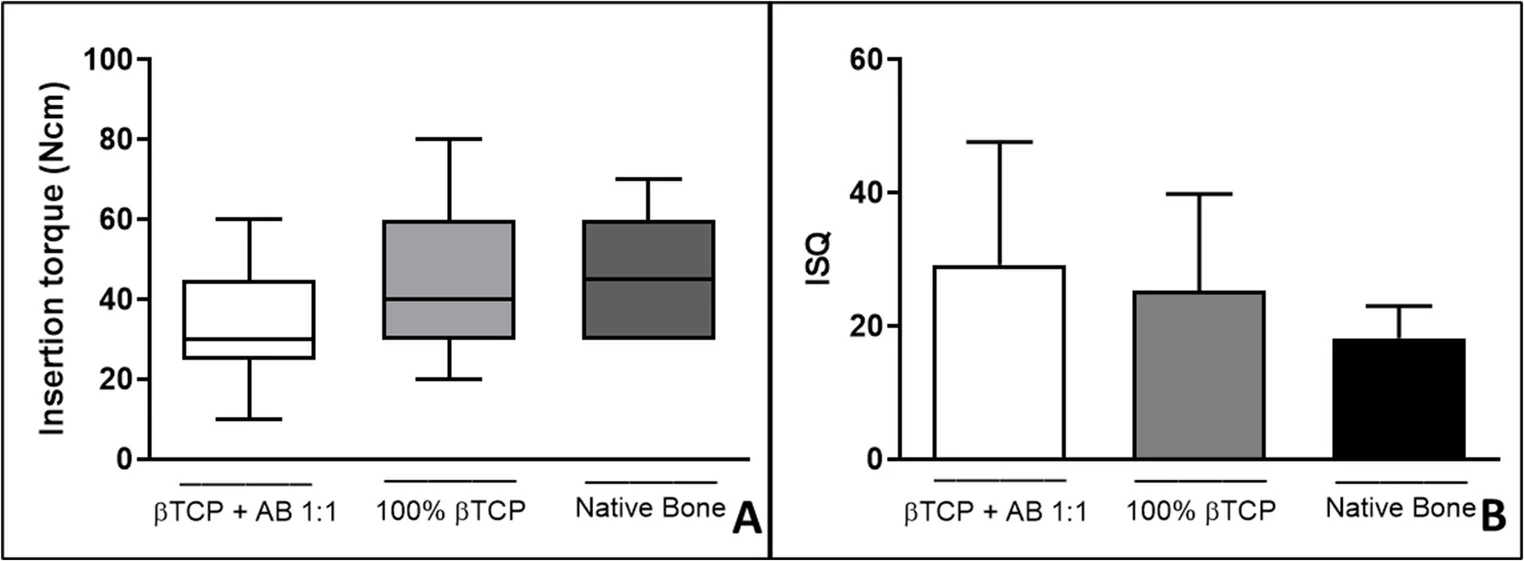
Box plot of mean implant insertion torque (**A**) and primary stability (**B**). Specifically, the mean insertion torque values were 37.71 ± 14.52 Ncm in areas grafted with β-TCP + AB 1:1, 41.88 ± 15.80 Ncm in areas grafted with β-TCP alone, and 46.82 ± 15.54 Ncm in native bone areas. Implants placed in areas grafted with β-TCP + AB 1:1 showed the highest primary stability values (28.50 ± 19.50), followed by implants in areas grafted with β-TCP alone (22.50 ± 12.50). Implants placed in native bone areas presented the lowest primary stability values (18.50 ± 4.50)

No significant differences were observed between the groups regarding implant insertion torque in grafted areas or native bone sites (37.71 [30.00] ± 14.52 Ncm for sites grafted with βTCP + autogenous bone 1:1; 41.88 [40.00] ± 15.80 Ncm for sites grafted with βTCP alone; 46.82 [45.00] ± 15.54 Ncm for native bone sites). Additionally, no significant differences were found between the groups in the resonance frequency analysis. Implants placed in sites grafted with βTCP + autogenous bone 1:1 exhibited primary stability values of 28.50 ± 19.50, implants placed in sites grafted with βTCP alone presented values of 22.50 ± 12.50, and implants placed in native bone sites presented values of 18.50 ± 4.50 (Fig. 2).

### Histomorphometric analysis

**Fig. 3:**
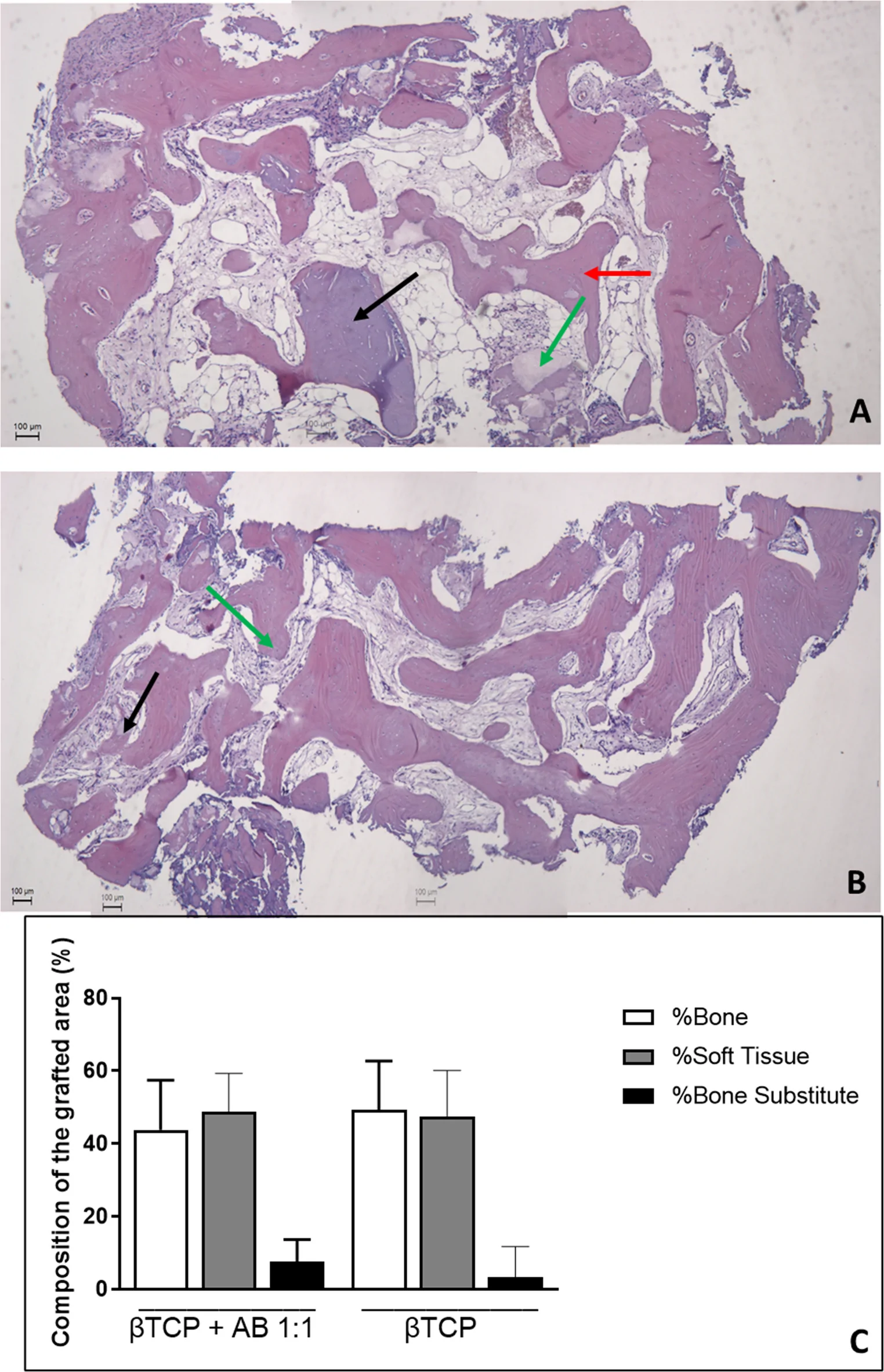
Histological sections of sinuses grafted with β-TCP + AB 1:1 (**A**) and with pure β-TCP (**B**), and box plot of grafted area composition (**C**). Sinuses grafted with β-TCP + AB 1:1 showed 43.75 ± 13.67% bone, 7.58 ± 6.03% residual bone substitute, and 48 ± 10.65% soft tissue. Sinuses grafted with pure β-TCP presented 49.32 ± 13.41% bone, 3.22 ± 8.46% residual bone substitute, and 47.45 ± 12.61% soft tissue

Regarding the composition of the repaired tissue, sinuses grafted with βTCP + autogenous bone 1:1 showed values of 43.75 ± 13.67% bone, 7.58 ± 6.03% remaining bone substitute, and 48 ± 10.65% soft tissue, and sinuses grafted with βTCP alone presented values of 49.32 ± 13.41% bone, 3.22 ± 8.46% remaining bone substitute, and 47.45 ± 12.61% soft tissue. No significant differences were observed between the groups in terms of the composition of the repaired tissue (Fig. 3). The bone tissue associated with the residual autogenous bone and β-TCP particles predominantly exhibited an immature morphology, with no clear evidence of complete lamellar maturation.

### Tomographic analysis

**Fig. 4:**
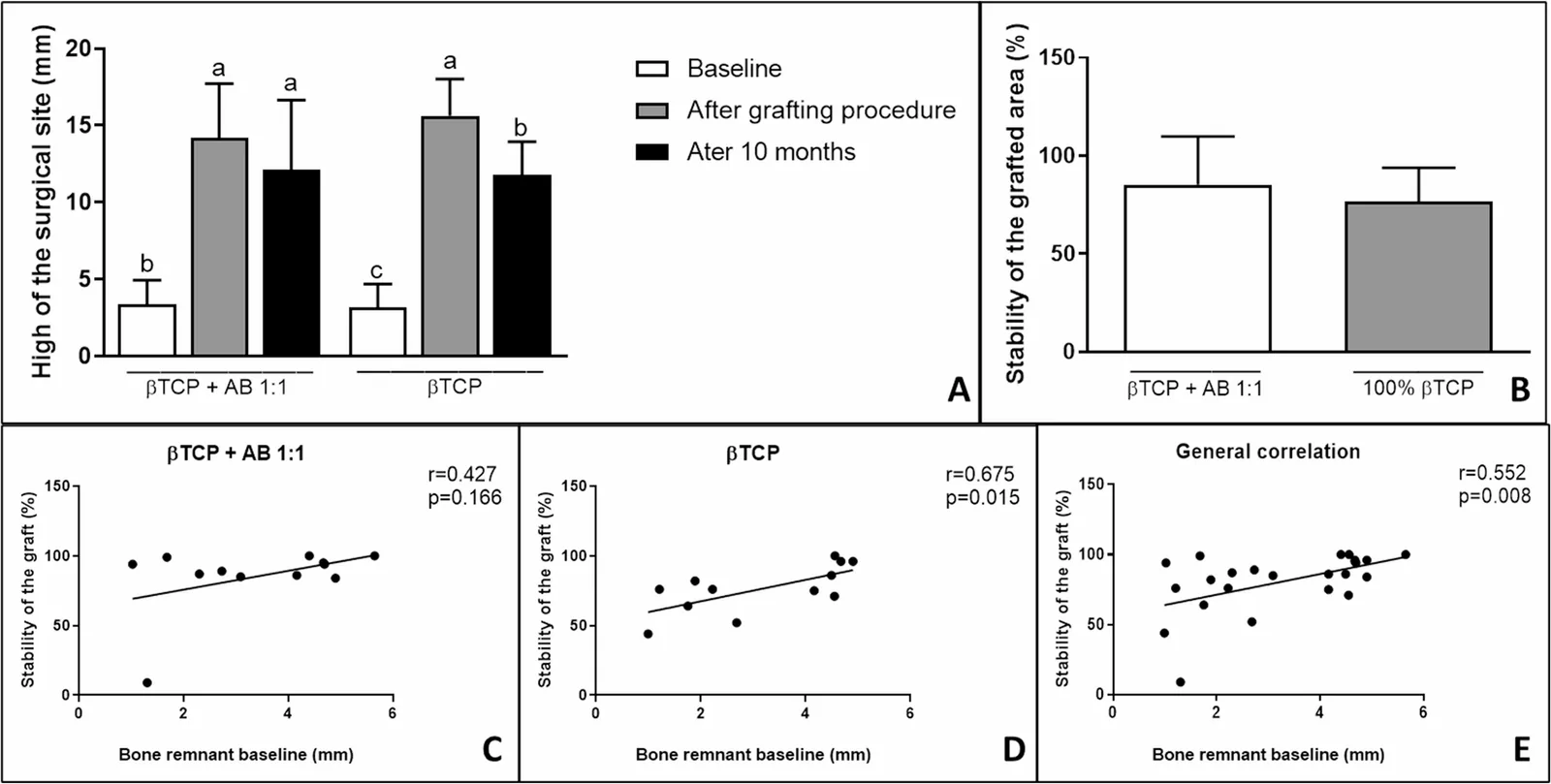
Evaluation of the linear height of the surgical bed at different time points (baseline, 14 days, and 10 months after surgery) in the groups grafted with β-TCP + AB 1:1 and β-TCP. Both groups showed a significant increase in surgical bed height after grafting, followed by a reduction at 10 months, with no significant differences between the groups. The maintenance of the grafted area height relative to the 14-day period was 85.17 ± 24.69% in the β-TCP + AB 1:1 group and 76.50 ± 17.27% in the β-TCP group. The correlation between the initial residual bone and graft stability was significant in the β-TCP group (*r* = 0.67; *p* = 0.01)

**Fig. 5:**
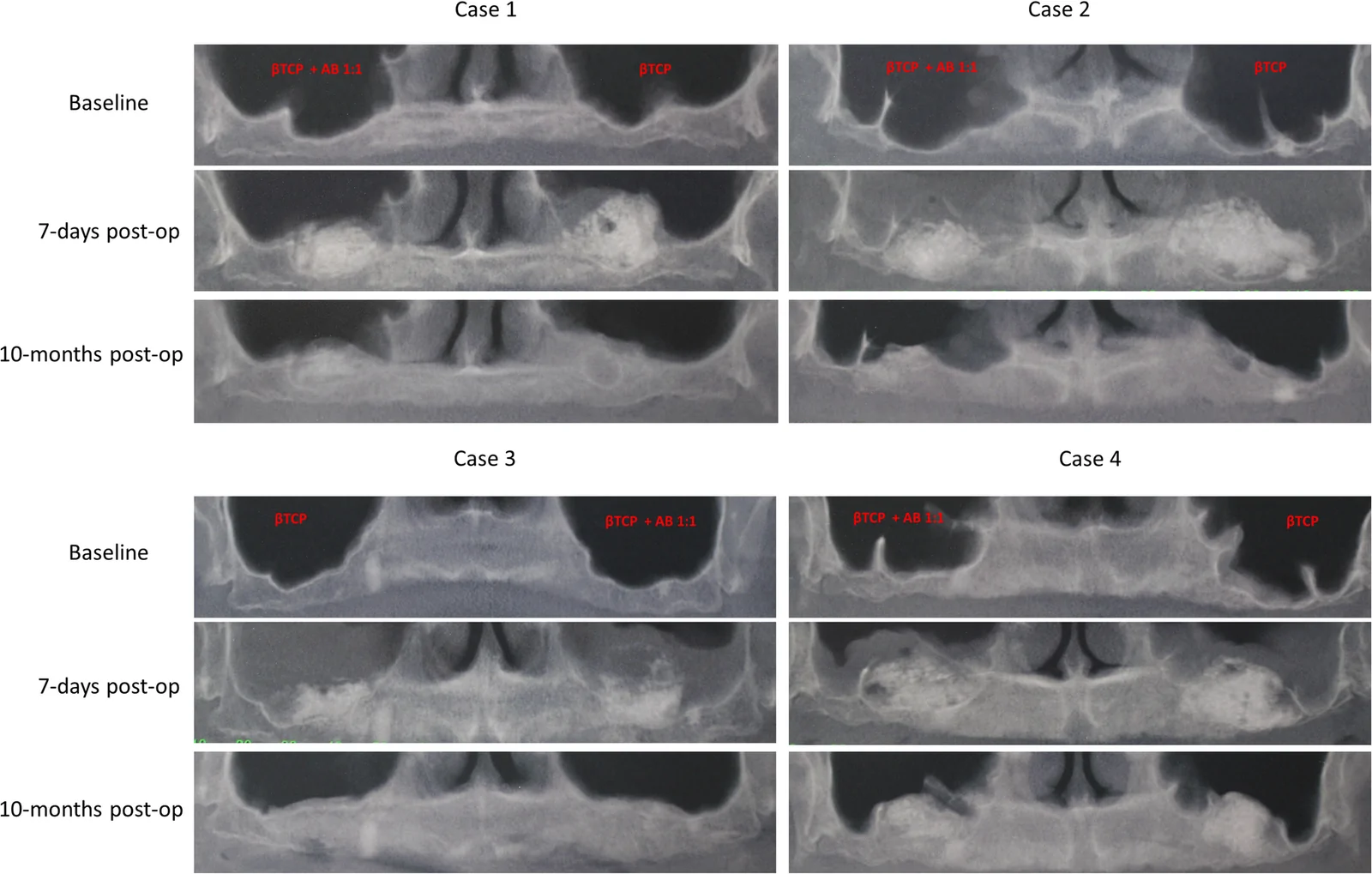
Representative panoramic images of the tomographic analyses of the groups at baseline, and 7 days and 10 months postoperatively

Both grafts promoted an increase in the height of the surgical bed where implants were subsequently placed. In the βTCP + autogenous bone 1:1 group, linear heights of 3.38 ± 1.56 mm at baseline, 14.18 ± 3.54 mm at 14 days post-surgery, and 12.12 ± 3.54 mm at 10 months post-surgery were recorded, while in the βTCP-only group, linear heights of 3.17 ± 1.51 mm at baseline, 15.62 ± 2.41 mm at 14 days post-surgery, and 11.81 ± 2.12 mm at 10 months post-surgery were observed. However, no significant differences were found between the groups regarding this parameter. At 10 months, the βTCP + autogenous bone 1:1 group maintained values of 85.17 ± 24.69% of the grafted height relative to the 14-day measurement, whereas the βTCP group maintained 76.50 ± 17.27% of the grafted height. The amount of initial bone influenced overall graft stability (*r* = 0.55; *p* = 0.008); however, this correlation was statistically significant only in sinuses grafted with βTCP (*r* = 0.67; *p* = 0.01) (Fig. 4). Representative images of the tomographic scans obtained before, 14 days after, and 10 months after the maxillary sinus floor elevation procedure are shown in Fig. 5.

## Discussion

The treatments evaluated in the present study were equally effective in increasing the amount of available bone tissue, allowing the placement of implants with satisfactory primary stability. Furthermore, the composition of the grafted areas and the stability of implants placed in these sites did not differ between groups. However, the β-TCP grafted areas exhibited a greater volume reduction than the areas grafted with β-TCP combined with autogenous bone specially in more atrophic sinus. Accordingly, the null hypothesis of this study, which stated that the addition of autogenous bone to β-tricalcium phosphate (β-TCP) would not influence healing outcomes, was partially rejected.

Tomographic analysis revealed a substantial gain in mineralized tissue following maxillary sinus floor elevation in both groups, supporting the predictability of this technique in achieving successful increases in bone availability [8, 23, 24]. A reduction in the volume of the grafted area was also observed 10 months after the surgical procedure. Indeed, previous clinical studies reported considerable volumetric resorption in maxillary sinuses grafted with particulate autogenous bone (e.g., 24–45% at 6 months post-surgery) [25, 26], as well as in areas grafted with β-tricalcium phosphate, which on average showed approximately 20% greater volume loss at 6 months post-surgery [13]. The association of the bone substitutes and autogenous bone grafts presented higher resorption, related to higher rates of autogenous bone graft [16], and the proportion of 1:1 was related to a 23% reduction in grafted area. Data presented by previous studies are in line with our findings, showing reductions in the volume of the grafted area within the range of 15–25%Some other factors, such as the healing period, the size of the grafted sinus, the purity of β-tricalcium phosphate, and the source of autogenous bone, may contribute to variations in volumetric resorption between sinus floor elevation and subsequent implant placement. Indeed, in our study, larger maxillary sinuses were associated with higher levels of grafted volume loss. These findings highlight the need for careful planning and the use of larger amounts of graft material in wider maxillary sinuses. However, caution is warranted regarding the excessive use of bone substitutes in the maxillary sinus, as greater distances from the grafted area to native bone are associated with lower new bone formation, regardless of sinus size [27, 28]. Furthermore, it is noteworthy that in the current study, sinuses grafted exclusively with β-TCP showed a greater reduction in graft volume compared to those in which β-TCP was combined with autogenous bone. It has been reported that β-TCP exhibits higher resorption rates than other types of osteoconductive bone substitutes, such as synthetic or natural hydroxyapatite [29]. These higher resorption rates of β-TCP may also explain the differences between the present findings and those of other studies, which demonstrated that the use of autogenous bone grafts increases grafted volume loss in direct proportion to the amount of autogenous bone relative to the total graft material volume. [16, 26]. In addition, it is important to note that the autogenous bone used in the current study was harvested from the posterior region of the mandible, which, due to its highly cortical nature, shows less volumetric loss compared to more trabecular donor sites, such as the iliac crest [30]. Interestingly, graft stability was significantly correlated with the initial residual bone height only in the β-TCP-alone group. This finding suggests that the dimensional stability of β-TCP may depend more strongly on the amount of residual native bone available at baseline. Therefore, although β-TCP alone resulted in similar histological outcomes after 10 months, its clinical performance may be less predictable in severely atrophic maxillary sinuses, where the addition of autogenous bone could contribute to maintaining graft volume during healing.

The grafted areas in the present study showed bone formation above 40% in both groups, with a similar amount of soft tissue and a low proportion of residual bone substitute particles. This tissue composition pattern is closely related to volumetric loss, as materials or associations of materials that induce greater formation of bone and soft tissues, with a lower proportion of residual bone substitute particles, tend to result in volume reduction [31, 32]. Another notable finding in this study was the lack of additional benefits from combining autogenous bone grafts with β-TCP compared to the use of β-TCP alone regarding the histological bone formation after 10 months of follow-up. The autogenous bone graft has been considered the best bone graft, as confirmed by previous studies. Systematic reviews showed that pure autogenous bone grafts exhibited a higher mean percentage of mineralized bone compared to substitutes or mixtures, demonstrating that autogenous bone may promote more pronounced histomorphometric outcomes. However, this advantage is accompanied by greater volumetric resorption and morbidity associated with donor site harvesting [31, 33].

Due to these inherent limitations, combinations of bone substitute materials with autogenous bone have been proposed, as exemplified in the present study. These combinations aim to reduce donor-site morbidity, while ensuring adequate bone formation and volumetric stability [11, 34]. This approach has previously demonstrated favorable outcomes, with enhanced bone formation and lower volumetric loss compared to the use of autogenous bone grafts alone [34, 35], findings that are consistent with those observed in our investigation. Previous studies reported that mixtures of autogenous bone and deproteinized bovine bone tend to promote greater bone formation as the proportion of autogenous bone increases [33, 36, 37]. However, these results remain controversial, as other investigations showed that the combination of autogenous bone with deproteinized bovine bone and β-TCP does not confer additional advantages in bone formation when compared with grafted areas treated with these materials individually [37, 38]. Moreover, clinical studies demonstrated that the survival and success rates of implants placed in maxillary sinuses grafted with deproteinized bovine bone, with or without the addition of autogenous bone, were comparable after 1–5 years of functional loading, [39–41]. Only a limited number of studies have assessed the combination of autogenous bone with β-TCP [11, 26, 37], and in all of these, the use of autogenous bone did not provide significant benefits regarding bone formation or volumetric maintenance of the grafted area compared with the use of β-TCP alone.

In the present study, implant insertion torque values were equal to or higher than 30 Ncm, whereas the ISQ values ranged between 30 and 40, which are considered low. The absence of significant differences between the groups can be explained by the fact that both received grafts with similar compositions, resulting in comparable bone characteristics at the implant sites. Moreover, insertion torque values above 30 Ncm indicate that, even in grafted areas, the implants achieved satisfactory mechanical retention, which may be associated with the presence of peripheral native bone. On the other hand, the low ISQ values can be attributed to the predominance of regenerated bone in the region, which, although capable of providing initial stability, exhibits lower rigidity compared to native bone. This occurs because resonance frequency analysis assesses not only the cortical bone stability at the time of implant placement but also the condition of the bone surrounding the entire implant [42]. Therefore, when part of the implant is surrounded by newly formed bone or by bone substitute material still undergoing remodeling, ISQ values tend to be lower, even when insertion torque is considered clinically acceptable [43].

The macrogeometry of the implants used in this study may also influence the primary stability data. Conical implants tend to engage more tightly during placement, due to the lateral compression of cortical bone, resulting in higher insertion torque values compared to cylindrical implants [44, 45]. This effect occurs because conical implants are designed to provide good initial stability even in low-density bone [46]. In the context of grafted areas, where regenerated bone presents lower rigidity and density, the conical design may compensate for these less favorable conditions, by improving mechanical interlocking with the surrounding bone. Although a substantial portion of implant stability depends on the grafting material, conical implants typically achieve adequate torque values (≥ 30 Ncm) even under these circumstances, reinforcing their suitability for use in reconstructed maxillary sinuses or areas augmented with bone substitutes. In the present study, despite satisfactory and comparable insertion torque values between groups, ISQ values were low, as abovementioned. This finding indicates that good mechanical engagement, partly provided by the conical design, does not necessarily translate into higher rigidity as measured by resonance frequency analysis [44]. These findings confirm that although the conical implant design contributes positively to insertion torque and initial mechanical stability, these gains may not be fully reflected in ISQ values, particularly in grafted areas.

Previous studies have already demonstrated that β-TCP yields favorable outcomes when used in grafting procedures for maxillary sinus floor elevation [11, 26, 47]. The main strength of the present study is that, to the best of our knowledge, it is the first split-mouth study to demonstrate that the addition of autogenous bone (AB) does not provide higher bone formation than in maxillary sinuses grafted with β-TCP after 10 months of healing. This finding is particularly relevant because the severe maxillary atrophy observed in the patients included in this study limited the use of more conservative autogenous bone harvesting approaches, such as obtaining bone from the maxillary tuberosity or from the lateral window created during sinus access. Therefore, the posterior mandible was selected as the donor site to provide the standardized amount of autogenous bone required by the surgical protocol adopted in this study [48]. Indeed, this procedure is associated with additional morbidity, including postoperative edema and trans- and postoperative discomfort. Therefore, the present findings are clinically important, as they suggest that the addition of AB did not increase the amount of newly formed bone after 10 months in this sample, even in more challenging cases requiring maxillary sinus floor elevation.

Among the limitations of the present study, the 10-month follow-up period between the grafting procedure and implant placement should be highlighted. Although this interval was longer than that reported in similar studies—which generally adopt a 6-month period—the decision was justified by the use of a graft material that has been clinically less explored, thereby warranting an extended observation time to ensure adequate integration. Although the addition of autogenous bone did not result in greater bone formation in the β-TCP-grafted areas after 10 months, it is possible that shorter evaluation periods could reveal a faster pattern of bone formation [49, 50]. Such findings could potentially reduce the waiting time required before implant placement. However, this aspect could not be assessed in the present study and should be investigated in future studies with earlier follow-up intervals. Another limitation is that the secondary stability of the dental implants was not assessed stability at the time of prosthetic delivery. In addition, no clinical follow-up of the implants was conducted after prosthetic rehabilitation, which precludes the evaluation of long-term parameters, such as the maintenance of stability, bone remodeling, and functional success rates. Another important aspect to consider is that part of the implant placement sites involved native bone and were therefore not exclusively composed of grafted bone. Consequently, the stability values observed in the current study reflect the combined contribution of both regions rather than the grafted area alone. Finally, it should be noted that the maxillary sinus represents a highly favorable environment for bone formation; thus, the findings of this study may not be directly applicable to other, more challenging clinical situations—such as horizontal ridge augmentation in severely atrophic alveolar ridges.

## Conclusion

Considering the findings of the present study, the addition of autogenous bone to β-tricalcium phosphate (β-TCP) in maxillary sinus floor elevation did not enhance histological bone formation after 10 months of healing compared with β-TCP alone. However, the stability of β-TCP alone appeared to be influenced by the initial residual bone height, with lower residual bone height being associated with greater graft volume reduction. Therefore, β-TCP alone may represent a viable alternative after prolonged healing, whereas the potential advantage of autogenous bone in cases with limited residual bone height or during earlier healing periods remains to be investigated.

## Data Availability

The data will be available upon request to the authors.

## Declarations

### Clinical trial registration

The study protocol was registered in the Brazilian Clinical Trials Registry (ReBEC – RBR-7qhkrn) on November 11, 2016, under the WHO Universal Trial Number (UTN) U1111-1173-6057.
